# Reduced Gaze Following and Attention to Heads when Viewing a "Live" Social Scene

**DOI:** 10.1371/journal.pone.0121792

**Published:** 2015-04-08

**Authors:** Nicola Jean Gregory, Beatriz Lόpez, Gemma Graham, Paul Marshman, Sarah Bate, Niko Kargas

**Affiliations:** 1 Psychology Research Centre, Faculty of Science and Technology, Bournemouth University, Poole, Dorset, United Kingdom; 2 Department of Psychology, University of Portsmouth, Portsmouth, Hampshire, United Kingdom; University of Lincoln, UNITED KINGDOM

## Abstract

Social stimuli are known to both attract and direct our attention, but most research on social attention has been conducted in highly controlled laboratory settings lacking in social context. This study examined the role of social context on viewing behaviour of participants whilst they watched a dynamic social scene, under three different conditions. In two social groups, participants believed they were watching a live webcam of other participants. The socially-engaged group believed they would later complete a group task with the people in the video, whilst the non-engaged group believed they would not meet the people in the scene. In a third condition, participants simply free-viewed the same video with the knowledge that it was pre-recorded, with no suggestion of a later interaction. Results demonstrated that the social context in which the stimulus was viewed significantly influenced viewing behaviour. Specifically, participants in the social conditions allocated less visual attention towards the heads of the actors in the scene and followed their gaze less than those in the free-viewing group. These findings suggest that by underestimating the impact of social context in social attention, researchers risk coming to inaccurate conclusions about how we attend to others in the real world.

## Introduction

In an ever changing environment, it is essential that we have some way of processing only the visual information with greatest relevance for us at each particular moment. Our eyes make around three movements per second and take in visual information with each intervening fixation. The question of how the eyes, and therefore the attention system, select the next target for fixation has been much debated [1–5]. Whilst the inherent visual properties of the scene are greatly important in this decision making process [6,7], the social content of the scene plays a critical role in directing our eye movements and attention.

Much work has demonstrated that when people are presented with photographs of scenes containing people, visual attention, as measured by eye movement behaviour, is readily directed towards the people in the scene, particularly their faces and eyes over and above their bodies and non-social objects [8–12]. This phenomenon is widely referred to as *social attention*. The term social attention is often also used to describe another way in which our attention system responds to the people around us, namely, the tendency to follow other peoples’ gaze. By looking where our social partners look, both gazer and responder are able to engage in *joint attention*, to allocate their attention to the same object or location, an ability which is critical to typical social development and which starts to emerge at the end of the first year of life [13–17]. The gaze following mechanism has been largely studied using the gaze-cueing paradigm, an adaptation of the Posner’s [18] spatial cueing paradigm. In this task, the typical finding is that lateral targets appearing on the same side as that being gazed at by a centrally presented face are quicker to be detected (via covert attention) [19–22] or saccaded towards (via overt attention) [23–25] than those appearing in the opposite direction. This finding is interpreted as the gaze cue causing a shift of attention in the observer in the direction corresponding to that of the cue. The effect has been shown to be modulated by factors such as the expression of the face [20,26,27], the task instruction [28,29], gender of the observer [30] and even the political leanings of the participant [31,32]. But the general consensus on the matter appears to be that the basic gaze orienting mechanism is reflexive (although see [33]) with some authors even suggesting it may be an innate mechanism [34].

Despite the fact that our social interactions tend to occur embedded within complex, dynamic social environments, it is interesting to note the vast majority of accumulated knowledge on social attention to date has arisen from highly controlled and constrained, laboratory-based studies, where participants are shown static images of faces or people in isolation whilst they have their eye movements recorded. That such studies lack in ecological validity is an argument which has begun to be levelled at this research in recent years. Specifically, critics [11,35,36] suggest that by including only static images of isolated faces or people in their paradigms, researchers may be failing to tap into genuine social processes (see [37,38] for discussions of the role of ecological validity in social attention research). Findings from the literature on autism support this possibility. Autism is characterised by, amongst other things, atypical responses to social stimuli [39] and one of the earliest behavioural indicators of a later diagnosis is a failure to engage in joint attention, a component of which involves responding to others’ gaze direction [40]. However, despite continuing to show atypical responses to social stimuli in everyday life, in some lab-based social attention studies individuals with autism present typical patterns of visual attention towards people and their faces [9,10,41] and are as influenced by gaze direction as individuals without the disorder [10,42–44]. Hence, at least in the case of autism, there may be sensitivity to the lack of genuine social involvement which is inherent in such lab-based tasks.

More recently, some researchers have attempted to address the lack of ecological validity of stimuli in social attention studies by presenting more complex, naturally occurring static scenes to participants whilst recording their eye movements [8,9,11,45–47] but there have been fewer researchers who have explored social attention within dynamic social scenes [48–51,51]. One group of researchers in particular have examined social attention, including gaze following behaviour, in naturalistic dynamic scenes. In their studies, Kuhn and colleagues [50–52] played recordings of a magic trick to participants, where an item (either a ball, cigarette or lighter) appeared to vanish from the hands of the magician. The vanishing illusion stems from observers following the magician’s gaze direction which strategically misdirects the attention of the observers away from the critical events of their trick. Kuhn and colleagues confirmed this to be the case using eye tracking as they found that participants’ gaze was shifted in the direction of the magician’s and away from the to-be-concealed event. These experiments provided the first evidence that gaze following occurred when watching dynamic social stimuli.

Thus far, the discussion of the ecological validity has focused on the degree to which the stimuli have corresponded to those occurring in the natural environment. However, that is not the only aspect of previous research that has been lacking in this respect. It has only been very recently that some have started to acknowledge that the context within which the behaviour is observed can influence visual attention. Never has this been more important than when it relates to social behaviour. As a pertinent example, Risko and Kingstone [53] showed that when participants believed that the eye tracker they were wearing was switched off, they were more likely to look towards a sexually provocative stimulus than if they believed the eye tracker was turned on. Thus, simply believing that they were engaged in a social scenario was enough to alter social attention. Thanks to advances in eye tracking methodology, it is now possible to measure visual attention in entirely natural environments and compare this to when the same scenario is played back to participants via a video monitor. Several studies have shown that in terms of general viewing behaviour, people in live social interactions attract as much attention as when they are presented on a video screen [49,54]. However some differences do seem to exist. One emerging theme is that participants, rather than follow gaze, appear to avoid the gaze of others when in close proximity to them. Laidlaw et al. [55] showed that when a participant was seated in a waiting room with an unknown confederate, they tended to look less at the confederate when they were physically present in the room, than when a video of the same confederate was played back on a monitor in an otherwise identical setup. Furthermore, Foulsham and colleagues [49] reported that participants walking down a street directed fewer fixations towards unknown pedestrians walking close by them, than when a video of the same scenario was played back in the lab. However, in both of these studies, the possibility that participants were following eye gaze direction of the people in the scene, rather than just avoiding their gaze, was not explored. Conversely however, in a study by Freeth at al. [54] participants engaged in a live face to face interaction with an experimenter were more likely to engage in eye contact with them than when they watched the same scenario in a pre-recorded video. However, a critical difference between this and the two aforementioned studies was that in Freeth at al.’s live condition, participants were actively engaged in an interaction with the experimenter, not just observing a stranger from afar, the latter being a situation is which it would be considered inappropriate to stare at someone. This difference may account for the increased gaze allocation to face in this study’s live condition, whereby participants found themselves in a scenario in which it is perfectly appropriate to look directly at someone’s face and make eye contact with them. Thus, it appears that social attention is indeed modulated by the social context in which it occurs, but quite which conditions are important in effecting this, is still somewhat unclear. However the findings of Freeth et al. [54] suggest the degree of social engagement with people in the scene may be important in determining whether gaze is avoided or not.

The improvements in the ecological validity in social attention research in recent years are certainly laudable. However, an important issue has been overlooked in all of these studies: When comparing video stimuli that are known to be recorded to live, embodied social scenarios, it is not one, but two parameters which have been altered. That is, not only have the stimuli changed (from a video to a real scenario) but so too has the context in which that stimulus is presented (from lab with no possibility of a social interaction occurring, to a scenario where a social interaction is either occurring or is likely to occur). It is therefore not possible to say with any certainty whether differences in social attention noted in previous studies arise from changes in the stimulus or changes in the social context. A recent neuroimaging study suggests that context alone can alter neural activity in the social brain network. Redcay et al. [56] asked participants in an fMRI scanner to engage in a live social interaction with the experimenter. They were later played the same interaction back whilst still in the scanner, knowing that it was pre-recorded from their earlier conversation. Despite the stimuli being identical between the two conditions, the anterior cingulate cortex, ventral striatum and amygdala, all areas associated with social reward [57] were activated to a greater extent in the live condition than in the recorded condition. As in the aforementioned Risko and Kingstone [53] study, a change in belief about the social context is all that was required to modulate responses to that situation.

The current study set out to determine whether altering the belief participants have about the social context in which they view a dynamic social scene influences their allocation of attention within that scene as well as their gaze following behaviour. We played a short video of a waiting room scenario, where two actors sat quietly reading magazines and interacting with their mobile phones, save for occasionally shifting their gaze towards anticipated events at the periphery of the scene. Three groups of participants were given different instructions before watching the video. In a baseline condition, participants simply free-viewed the video with no instruction, save to watch the video until it ended. In two “social” conditions, participants watched the video with the belief that they were viewing a live webcam of other participants who were taking part in the study. In the first social group, the “socially-engaged” group, participants were further told that they would be taking part in a group task with the participants in the video and would be taken through to meet them once they had been given the opportunity to view them via the webcam. In the other social group, the “socially non-engaged” group, participants were informed that they would perform a task alone, rather than with the people in the video, and that they would at no point meet them. We embellished the social scenario further in these two conditions by involving another experimenter who was “greeting the other participants” in the waiting room, and who entered the lab to advise when to begin watching in the “webcam”.

We predicted that as the social relevance of the scenario increased from free-viewing to socially non-engaged, to socially-engaged, we would notice corresponding changes in the visual attention of the participants to the people in the scene. The participants in the free-viewing group who knew the video to be pre-recorded should have felt free to fully explore the scene without the need to adhere to any social rules governing appropriate behaviour when in the vicinity of others, and as in previous studies, we expected a bias towards the people in the scene, particularly their heads, to emerge. In the social conditions, as the actors in the scene could not interact with the participants or see them, we anticipated that participants would also be free to fully explore the scene without any need to adhere to any social interaction rules (e.g. as not staring at strangers) which are likely to be responsible for the gaze avoidance behaviour observed in previous tasks using live social scenarios. However, we also predicted that the social context in these two groups would increase the social salience of the actors in the scene and therefore we predicted more fixations to heads and bodies than in the free-viewing group., We further predicted an enhanced bias toward heads and gaze following in the socially-engaged group based on two theoretically motivated reasons. First, we expected the social relevance of the actors to be particularly pronounced in the socially-engaged group, as participants believed they would eventually interact with the actors. Work by Smilek et al.[11] and Birmingham et al. [45] [11,45] has shown that when people are asked to make mental state inferences about individuals depicted in social scenes, participants spend longer looking at their heads than when they free-view the same scenes. Therefore, we anticipated that participants in the socially-engaged condition would spend more time looking at the heads of the actors and be more likely to follow their gaze than in the other conditions as they would be more motivated to make judgments about the personalities and mental states of the people they were about to work with than those who did not expect to meet the actors. Secondly, social identity theory [58] would predict that the actors in the scene would constitute in-group members to the participants, as they were about the complete a group task with them and would therefore be likely to share a common social identity. Very recent work by Kawakami et al. [59] has demonstrated a visual attention bias towards the eyes of others who are members of the same social group when compared to those who are out-group members. Increased attention to heads in general and particularly the eyes could be logically expected to result in increased gaze following, as the gaze shifts would be more visually apparent. As the social relevance of the actors in the scene would be less pronounced in the non-engaged group where participants did not expect to meet the actors, we expected that participants would be less inclined to spontaneously form impressions about the actors, and therefore to look less at heads and gaze follow less because of this, than those in the socially-engaged group. The actors would also be less likely to be categorised as in-group members by the non-engaged group participants as they did not expect to complete a task with them, further supporting a prediction of a reduction in dwell time to heads and gaze following, relative to the engaged group.

As no previous study has examined dynamic gaze following in a setup such as the one used is the current study, it was difficult to make precise predictions about gaze following behaviour. The most comparable study to the one presented here has shown that when viewing a magic trick, participants spend around 20% more time looking at the hand a magician is looking at than the hand he is not looking at[51]. Also, in a study using static scenes, 14% of saccades made from the head of the person in the scene landed on the object the person was gazing at [47]. What both these studies suggest is that although gaze following seems to be a robust phenomenon, gaze cues are only one element of a scene impacting on attention allocation. Therefore we might expect a similar attentional advantage for gazed at targets here; that is, a modest but significant attentional bias towards objects gazed at by the actors. As with general gaze behaviour we expected an enhanced bias to follow gaze with increased levels of social engagement, as the actors in the scene became more socially relevant to the participants.

## Materials and Methods

### Participants

Eighty four participants took part in the experiment in exchange for £5 or course credit. Participants were students and staff from Bournemouth University and the University of Portsmouth, UK. Participants were recruited from two different sites due the experimenter changing institutions during the study. All participants had normal or corrected-to-normal vision. Six participants were excluded due to poor calibration of the eye tracker. A further participant was excluded as they disclosed that they had Asperger’s Syndrome and another participant was excluded as it transpired that they had misunderstood the experimenter’s explanation of the task. A post-experiment manipulation check resulted in a further four participants in the socially-engaged group being excluded due to them not believing that they would meet the actors in the video. One participant was similarly removed from the non-engaged group as they did believe they would meet the actors in the video, despite being told that they would not. This resulted in a final sample of 71 participants of which 60 were female, with an age range of 18–60 years old (M = 25.62 years, SD = 9.72).

### Ethics Statement

All participants gave full written consent and the experiment was approved by the ethics committees of the Psychology departments of both Bournemouth University and the University of Portsmouth. The individuals who acted in the video scene presented in this manuscript have given written informed consent (as outlined in PLOS consent form) to publish images taken from this video.

### Apparatus and Materials

Participants at both sites were tested using an identical eye tracker: the Eyelink 1000 with desktop mount (SR Research, Canada). Participants sat 57cm form the eye tracker with their heads stabilised by the use of a chin rest. At the University of Portsmouth, the eye tracker was connected to an SR Research Eyelink host computer, which was in turn connected to the display computer which was a Dell T3400 with a 19” Viewsonic G90B CRT monitor. At Bournemouth University, the eye tracker was connected to a Dell Optiplex 760 host computer connected to a HP Compaq dc7800 display computer and a 22” ProNitron 21/750 CRT monitor. Pupil and corneal reflection position were recorded monocularly at a rate of 1000Hz. Saccades are parsed online by the Eyelink 1000 using a velocity threshold of 30°/s and an acceleration threshold of 8000°/s^2.^

**Fig 1:**
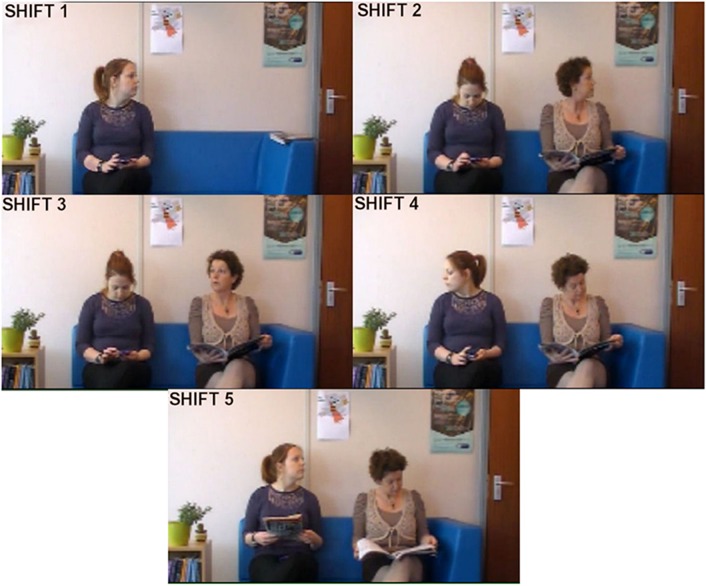
Screen shots of the five gaze shifts occurring in the video. The individuals who acted in the video scene presented in this manuscript have given written informed consent (as outlined in PLOS consent form) to publish images taken from this video.

The video stimulus was filmed in a seminar room at the University of Portsmouth using stationary digital video camera at a rate of 30 frames per second at a size of 720 x 400 pixels. The audio track was removed from the edited video. The video was initially prepared using Adobe Premier 9.0 and the quality was deliberately degraded to give the appearance of a live webcam stream. In order to create the experimental programme, the video was converted to an. xvd file using SR Research’s Experiment Builder software. The first frame of the video showed a female actor sitting on a sofa in a waiting room scenario whilst interacting with her mobile phone. To the left of her, a bookcase was visible and to the right of her was an empty space on the sofa. Further to the right, an open door was partially visible. Two posters were visible on the wall behind the chairs. After approximately 10 seconds, a second female actor entered the room and sat next to the first actor after extending a short greeting. The actors remained seated throughout the video, which lasted for two minutes, either interacting with their mobile phone or reading a magazine. On five occasions during the video, a gaze shift (head turn and eye gaze together) towards an object or event in the room was performed by one of the actors. The gaze shifts always began before the target of the shift was apparent. In three shifts, the actors looked towards the open door as if they heard the approach of another person. In another shift, the actor looked towards the bookcase. In a further shift, the actor looked towards an out of shot actor as if they had attracted their attention by moving suddenly. A period of between 1.8 and 3.2 seconds elapsed before the target of the shift became apparent. In the case of the shifts towards the door, the target event was someone entering the room, with just their hand entering the shot for a moment as they walked through the door. In the case of the shift towards the off-screen actor, the target event was a magazine being turned by that actor which briefly entered the shot on one side of the screen. In the case of the bookshelf shift, the target event was slightly different to the others in that the actor reached out and picked up a magazine which had attracted her attention. After approximately 1.5 minutes into the video, one actor asked the other the time. Although this technically involved gaze shifts from both actors, it was deemed to be so qualitatively different to the other shifts (which involved a gaze shift of a solitary actor towards an unknown target) that it was considered inappropriate to include this period in the gaze shift analyses. The five gaze shifts can be seen in Fig. 1.

### Procedure

For the two social conditions (engaged and non-engaged), the study was advertised as a “social interaction” study where participants would be required to carry out a group task with others. On arrival, the participants in the socially engaged group were briefed that the study would involve them completing a series of problem-solving tasks with some other participants who were being greeted in another room of the building by the second experimenter at that very moment. In the non-engaged group, on arrival participants were told that although a group task was due to be conducted with some other participants who were arriving in another room, they themselves were not going to take part in this task and would not therefore be meeting the other participants. Rather, they would be completing the tasks alone. Participants in both groups were informed that a webcam had been set up to the other reception room. The participants were informed that they would be required to watch the webcam whilst the other participants arrived. The socially engaged group participants were told that the study was concerned with the influence that viewing the other group members before they met them would have on how they later performed the task with them. The non-engaged group were told that they would simply be required to watch the webcam of the other participants as part of the experiment. For both groups, whilst the participants completed the consent form, the experimenter pretended to check that the webcam was working, by bringing up a pre-recorded video of the first actor in the waiting room apparently talking to someone off screen. Whilst most participants failed to notice this on the screen, the manipulation was included in order to improve the authenticity of the scenario should the participant turn to look at it. The experimenter then explained to the participant that only one participant appeared to have arrived so far in the waiting room. The screen then turned black. Following this, the participant was seated at the eye tracker whilst the experimenter explained that they were waiting for the second experimenter to tell them that the second participant had arrived and were therefore ready to start. In order to further improve the authenticity of the scenario, after a few seconds, the first experimenter telephoned the second experimenter on her mobile phone in front of the participant to check whether it was possible to start. After a few seconds, the second experimenter knocked on the door and entered the lab, explaining that “one participant has arrived, but the other has just called to say she is just on her way now, so you may as well get started”. The eye tracker was then calibrated using a nine point calibration procedure. Participants were instructed to simply watch the “webcam” until they were told to stop by the experimenter. This was followed by a white message on the black screen reading “connect to webcam host? Y/N” for the experimenter to begin playing the video.

In the free-viewing group, the social pretext of other participants being present and a group task taking place, together with a live webcam into the waiting room was not included. Participants arrived at the lab and were greeted by one experimenter, were calibrated as above, and asked to simply watch a short video. The experimental programme was identical for all three groups.

Following the above between-group procedures, all participants completed a short drift correction procedure, which corrected for any eye drift subsequent to the calibration. The video was then presented centrally as a 720 x 400 pixel window on an otherwise black screen (NB: the video was displayed at the same resolution on both monitors used in this experiment) and lasted for two minutes. Eye movements were recorded for the duration of the video and timestamps were recorded to the data file for each frame of the 30 frame per second video. After this time, the screen turned black, save for a message in the top left hand screen, presented in white which read “connection to webcam host 192.162.3.1 lost” in order to maintain the authenticity of the social pretext in the engaged and non-engaged groups.

For the two social groups, at the end of the eye tracking phase participants were asked to fill in a short questionnaire which asked the extent to which they believed the webcam had been live and the extent to which they believed that they would meet the people in the video on a scale of 1 to 7 (1 = total disbelief to 7 = total belief). Participants were then debriefed as to the true nature of the study and the reason for the need for deception explained in full.

## Results

### Data Handling

**Fig 2:**
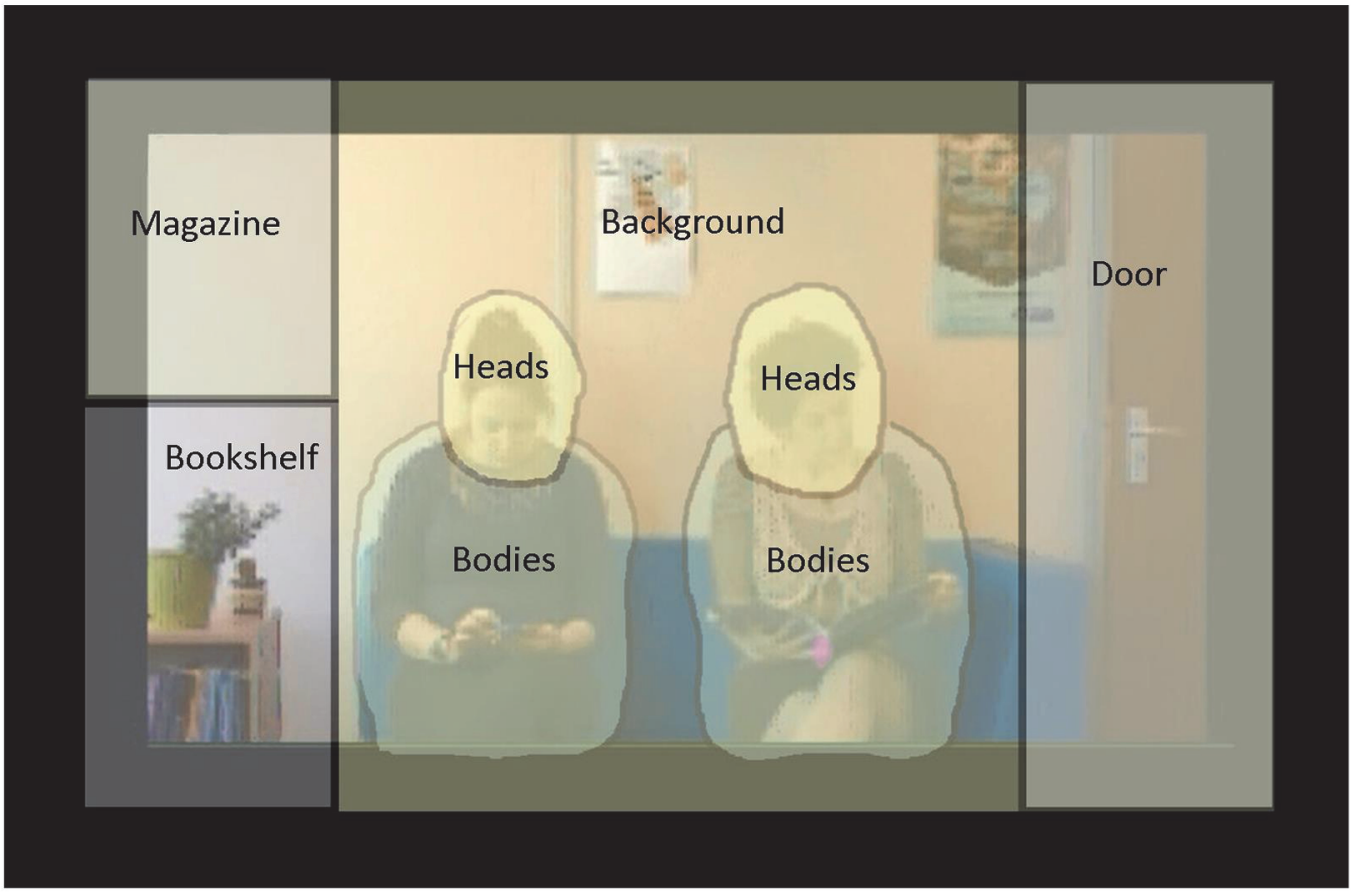
Interest areas (IAs) drawn using Dataviewer (SR Research) recreated in Corel Paint Shop Pro X for publication purposes. Note that for the general viewing behaviour analysis, the IA *Background* encompassed all IAs except the bodies and heads of the actors. Also note that a magazine being turned by an off-screen actor was present in the *Magazine* IA on one gaze shift only.

The actors moved very little during the critical periods of the video, save for the movements involved in the gaze shifts themselves and small movements of the body whilst turning pages of a magazine or interacting with a mobile phone. It was therefore possible to draw static interest areas around the important areas of the scene using Dataviewer (SR Research) which included small margins of approximately 20 pixels around the actors’ heads and bodies to allow for any movement that occurred during the gaze shifts. Aside from the interest areas drawn around the heads and bodies of the actors, for which the free-hand interest area tool was used, rectangular interest areas were drawn around the target areas of the scene, namely the bookshelf, the door and the area where the magazine briefly appeared when turned by an off-screen actor, as well as one large rectangular area drawn around the video window itself, which encompassed all elements of the scene. As the target objects were ambiguous and out of shot at the point of the gaze shift (e.g. person coming down corridor to eventually enter the door), the interest areas drawn around the eventual target areas were extended by a margin of 50 pixels in all directions to take into account participants’ uncertainty about the exact target of the gaze shift. The less desirable alternative was that these saccades would land in unanalysed areas of the screen, despite them clearly being the result of the gaze shift of the actor and therefore would be missed from the analyses. Interest areas can be seen in Fig. 2.

### General viewing behaviour

#### Proportion dwell time to interest areas

**Fig 3:**
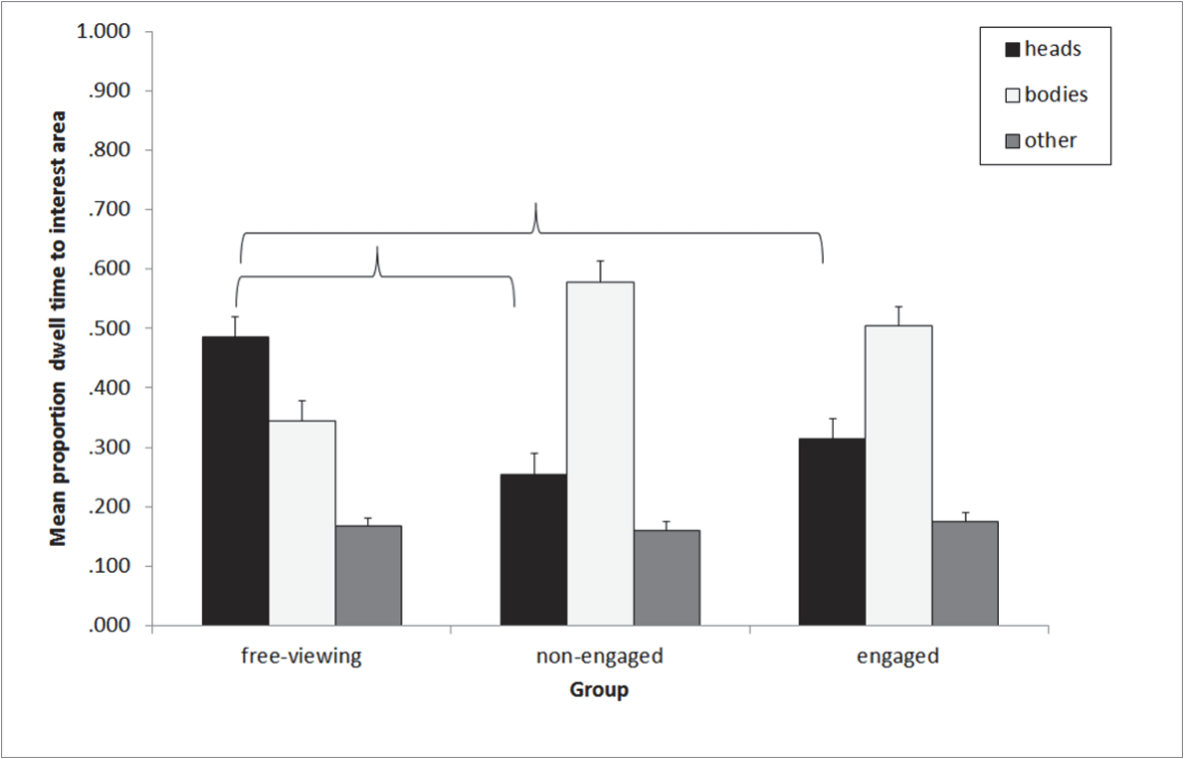
Mean proportion of dwell time to the heads, bodies and background of the scene across the three groups. Error bars represent standard error of the mean. Brackets indicate a significant difference at the p = .05 level.

An initial 2 way mixed- measures ANOVA was conducted on the proportion dwell time to the people in the scene and all other non-social objects across the three groups (free-viewing, engaged, non-engaged) during the full trial period from when both actors were present. The results confirmed findings of previous research showing that participants prefer to look at people in a scene than at non-social objects, regardless of group F_(1, 68)_ = 1557.93, p <. 001, η^2^_p_ = .958. There were no differences in this pattern across the groups, F < 1.00, p = .783. It was of importance for the current study to examine the relative dwell time allocated to the different social interest areas (heads and bodies of the actors). Therefore a second mixed-measures ANOVA was conducted with Group (free-viewing, non-engaged, engaged) as the between-participants factor and Interest Area (IA; heads, bodies, background) as the within-participants factor. Note that the IA “background” contained all of the video area except the people, including the target interest areas. A main effect of IA was found, F_(1.32, 89.84)_ = 56.29, p <. 001, η^2^_p_ = .453 (H-F criterion). Planned comparisons showed that participants spent significantly less time looking at the heads of the actors than at their bodies (p = .002) and significantly less time looking at the non-social elements of the scene than at heads (p <. 001) However, there were no differences in proportion of dwell time overall across the three groups, p = .305. There was a significant interaction between Group and IA, F_(4, 136)_ = 11.22, p <. 001, η^2^_p_ = .248. Planned comparisons across the groups showed that the free-viewing group spent significantly longer looking at the heads of the actors than the non-engaged group, p = .001, and the engaged group, p <. 001. There was no difference in the time spent looking at heads between the non-engaged and the engaged group, p = .217. Further comparisons between proportion of dwell time to bodies of the actors showed that the free-viewing group spent significantly less time looking at bodies than the non-engaged group, p <. 001, and the engaged group, p = .001. Again, there was no difference between the non-engaged group and the engaged group, p = .132. Further planned comparisons showed that there were no differences between the groups in time spent looking at the non-social elements of the scene, ps >. 400. The proportion of dwell time to the three IAs across the groups can be seen in Fig. 3.

### Gaze-following

In order to investigate the influence of the actors’ gaze shifts on the participants’ attention and eye movements, two measures of gaze following were calculated. The first examined overt gaze following, that is, the proportion of gaze shifts during the gaze shift period but before the target became visible, where a saccade started in the head IA of the actor making the shift and ended in the target interest area. The second measure examined a less direct influence of the gaze shifts on attention, by comparing the proportion of dwell time to gazed-at targets before and after the gaze shifts.

#### Overt gaze following

**Fig 4:**
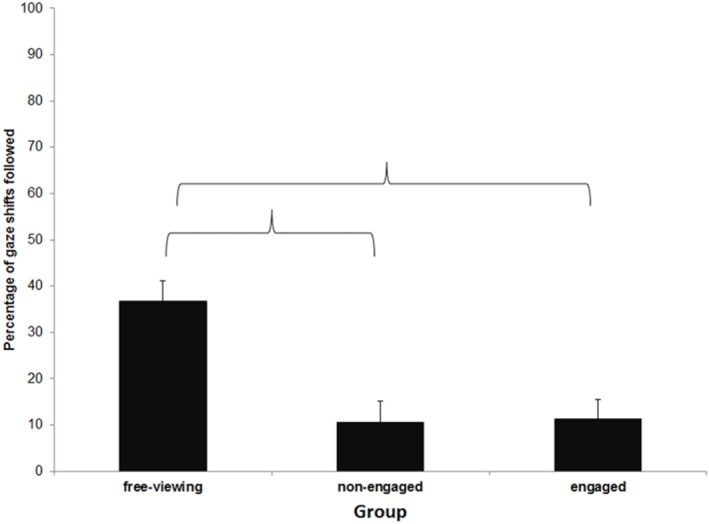
Mean percentage of gaze shifts followed in each condition, as defined by the proportion of shifts where a saccade starting from the head of the gazing actor landed in the target interest area. Error bars represent standard error of the mean. Brackets indicate a significant difference at the p = .05 level.

A one way between subjects ANOVA was conducted on this data and a main effect of Group was found, F_(2, 68)_ = 11.76, p <. 001, η^2^_p_ = .257. Planned comparisons showed that the free-viewing group followed significantly more gaze shifts (M = 36.80%, SD = 4.31) than either the engaged (M = 11.20%, SD = 4.31), p <. 001, or the non-engaged group (M = 10.48%, SD = 4.70), p <. 001. There was no difference between the two social groups, p = 0.910. Overt gaze following rates can be seen in Fig. 4.

#### Dwell time to gazed-at targets

A second measure was calculated in an attempt to capture less direct attention orienting caused by the shifts of gaze of the actors. It was anticipated that attention may be biased toward the gazed-at targets even if an initial fixation did not fall on the actor’s head during the shift. Therefore the mean proportion of dwell time on the gazed-at target IAs was calculated for the period of the gaze shift and the exact same duration immediately prior to the start of the shift for each of the five gaze shifts. This resulted in one pre- and one post-gaze shift period for each shift. For example, if the full duration of the gaze shift was 2000ms (the post-shift period), taken from the first frame where the actor began to turn her head to make the gaze shift to the first frame where the target appeared on the screen, the 2000ms immediately preceding that first frame of head movement was taken as the pre-shift period. Gaze shift durations ranged from 1800ms to 3200ms (M = 2360ms, SD = 577). In shifts 1, 2 and 5, the gazed-at target interest area was the door on the right of the scene where the hand of an off-screen actor (the target) appeared briefly as they walked through the door. In shift 3, the target interest area was a region where a magazine being turned (the target) by an off-screen actor briefly appeared in shot to the left of the scene. In shift 4, the target interest area was a bookshelf to the left of the scene which one of the actors turned towards to pick up a magazine. In this shift, the post-shift period was defined as from the first frame where the actor started to move her head to look at the bookcase, to the first frame where she began to move her hand to pick up the magazine. Dwell times were averaged across the 5 pre-shift periods and similarly averaged across the 5 post-shift periods. This procedure gave a measure of gaze-induced orienting, whereby dwell times immediately before the gaze shifts which fell in the target interest areas were compared to those falling in the target interest areas after the gaze shifts. This measure has similarities to the so-called gaze cueing effect reported in saccadic tasks based on the Posner-style spatial cueing paradigms [18,25,60]. The dependent measure in these tasks is the difference in saccadic reaction time towards congruent and incongruent gaze targets, a measure designed to identify biases of attention caused by the cue’s direction but in the absence of an overt gaze-following response.

A mixed-measures ANOVA was conducted on the mean proportion of dwell time allocated to the target interest area with two levels on the within-subjects factor Period (pre-shift, post-shift) and 3 levels on the between-subjects factor Group (free-viewing, non-engaged, engaged).

**Fig 5:**
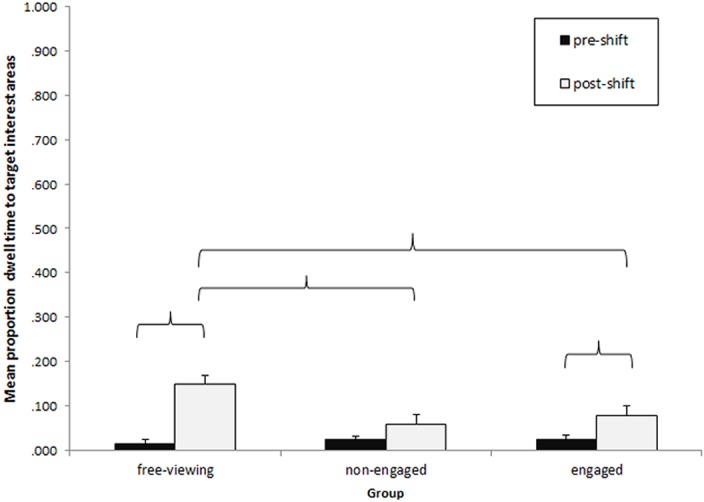
Mean proportion dwell time to the target interest areas before and after the gaze shifts across the three groups. Error bars represent standard error of the mean. Brackets indicate a significant difference at the p = .05 level.

A significant main effect of Period was found, F _(1,68)_ = 30.89, p <. 001, η^2^_p_ = .312, with greater proportion of dwell time allocated to the target interest areas after the gaze shifts than before the gaze shifts. There was a significant main effect of Group on dwell time to the targets overall, F _(1,68)_ = 3.38, p = .040, η^2^_p_ = .090, with the free-viewing group spending longer looking at the targets overall than the non-engaged group, p = .016, with a similar difference which approached significance in the engaged group, p = .066. There was a significant interaction between Period and Group, F _(2, 68)_ = 5.22, p = .008, η^2^_p_ = .133. Planned comparisons showed that both the engaged, p = .018, and the free-viewing groups, p <. 001, spent longer looking at the gazed-at target after the shift than before, but there was no difference between the two periods in the non-engaged group, p = .161 (Fig. 5). None of the other interactions approached significance, ps >. 130.

Further comparisons across groups showed that there were no group differences in time spent fixating the target IAs before the shifts, ps >.350. However, the free-viewing group spent significantly more time fixating the targets after the shift than both the engaged, p = .022 and the non-engaged group, p = .005. The difference between the two social conditions was not significant, p = .496.

### Relationship between Dwell Time to Heads and Gaze-Induced Orienting

The relationship between the two gaze orienting measures and time spent looking at the heads of the actors was examined to explore the possibility that participants who had longer dwell times to heads were also noticing the gaze shifts more readily and therefore responding to them to a greater extent. A bivariate Pearson’s correlational analysis was conducted between mean proportion of dwell time to heads and the mean gaze-orienting effect (mean difference in proportion dwell time to target IA pre- and post-shift). No significant relationship was found, r = -.054, p = .655. A second correlational analysis was conducted between the dwell time to heads and the proportion of gaze shifts overtly followed. Here, a significant positive correlation was found, r = .294, p = .013, demonstrating that the more time was spent looking at heads of the actor, the more gaze shifts were overtly followed.

### Correlations between Dwell Time to the Three IAs

Bivariate Pearson’s correlational analyses were conducted between proportion of dwell time to heads, bodies and background IAs. Dwell time to heads and bodies were strongly negatively correlated, r = -931, p <. 001, whereas no significant correlations existed between dwell times to either of these IAs and background (ps >. 110)

## Discussion

The aim of the current study was to examine the role of social context and potential social interactions on eye movement behaviour and gaze following when watching a video of a social scenario. In two social conditions, participants believed that the waiting room video was a live webcam feed of other participants waiting in an adjacent room with whom the participant would meet shortly to complete a group task (socially engaged group) or would not meet at all (socially non-engaged group). In a third condition, participants simply free-viewed the same video in the absence of any social pretext. Previous work [11,45] has demonstrated that when participants are asked to form impressions of the mental states of people depicted in photographs, they spend a greater proportion of time fixating their heads than when they are given no such instruction. Individuals have also been shown to allocate more fixation to the eyes of in-group members than out-group members [59]. We therefore hypothesised that gaze following and attention towards the heads of the actors would be greatest in the socially engaged group as participants would attempt to form an impression of the actors that they were due to meet and with whom they would take part in a group task. As the potential for social interaction decreased, and participants therefore became less socially-motivated in their viewing of the scene in the non-engaged group, we expected to see a reduction in gaze following and attention to heads compared to the engaged group. Finally, we predicted that the free-viewing group would show the least gaze following behaviour and attention to the heads of all three groups as participants in this group had no social involvement whatsoever with the actors in the video and no belief that the actors were present in the building.

The findings of the study entirely contradict these predictions. Rather than social involvement in the task increasing gaze following and attention to heads of the actors, participants in the two social conditions allocated significantly less visual attention to the heads of the actors than was seen in the free-viewing condition and in contrast, spent significantly longer time looking at the bodies of the actors than the free-viewing group. Similarly, overtly following of the actors’ gaze shifts was most pronounced in the free-viewing condition whilst there were no differences in overt gaze following between the two social groups. In addition, the free-viewing group spent longer looking at the gaze targets than the socially engaged group whilst the non-engaged group’s attention was not biased by the actor’s gaze cues. Although overt gaze following was associated with greater attention being paid to heads, dwell time to gaze targets was not related to the amount of time spent looking at the heads therefore it was not a simple case of the social groups noticing the gaze shifts less than the free-viewing group and this accounting for the differences in gaze orienting behaviour. In sum, the differences in gaze allocation in this study were found between the free-viewing group and the two social groups, whereas the gaze behaviour of the two social groups was virtually indistinguishable.

### Social Context and General Viewing Behaviour

The belief that the actors were being viewed in real-time and were present in the building radically altered participants’ visual attention and gaze following behaviour. Rather than enhancing the bias towards heads in social scenes, participants appeared to avoid the heads of the actors relative to free-viewing condition. Whilst our participants were not explicitly instructed to make any judgments about the mental states of the actors, we anticipated that our manipulation of the social context to include a future social interaction would result in participants considering the actors to be in-group members and to engage in a greater degree of mentalising, as they attempted to form perceptions of the individuals with whom they were to imminently interact [11,45]. Based on these assumptions, we would have expected participants in the engaged condition to allocate the most attention to heads than the other groups. However this was not the case. The reason for this may be apparent when more closely examining previous research in this area. Laidlaw et al. [55] demonstrated that when in the physical presence of a non-interacting confederate, participants are less likely to look at the confederate than when that confederate is presented as a pre-recorded video on a monitor within an otherwise identical scenario. Foulsham and colleagues [49] similarly showed that participants were less likely to fixate people who walked past them at close range whilst they walked along a street wearing a mobile eye tracker, than if the same scenario was watched on a video monitor in the laboratory. These findings suggest that the potential for a social interaction can cause attentional avoidance of others where a social interaction is possible or imminent. However, the crucial difference between these and the current study is that in the live conditions of the aforementioned studies, not only were participants able to view the protagonists in the scenes, the protagonists were also able to see the participants. Therefore, a two way interaction was possible at any moment and in such circumstances, implicit social rules would require participants not to visually engage with strangers they encounter. These social rules did not apply in the current study, as the actors could not see the participants, yet still participants displayed head-avoidance behaviour. However, when viewing what they knew to be a pre-recorded social scene and when there were no social rules to adhere to, participants in Foulsham et al. and Laidlaw et al.’s studies showed increased dwell time to the faces of the people within them, as was the case in the current study in the free-viewing condition. As there were no differences between the attention allocated to heads in the two social conditions in our experiment, it appears that the belief that the actors were fellow participants waiting in another room was enough to induce this head-avoidance behaviour, regardless of whether or not a live interaction with them was about to occur and regardless of the fact that the actors could not see the participants. It is unclear why this might be the case. One possibility is that the participants in our study suspected that the actors could also see them, as we did not explicitly state that they could not. Participants may therefore have been adhering to the “it’s rude to stare” social rule whilst they viewed the actors in the scene. An alternative explanation for this finding is that rather than the social groups avoiding the actors’ heads per se, it is possible that the other regions of interest commanded higher attentional priorities in these two groups than in the free-viewing group. Indeed, relative to the other conditions, there was a tendency for both social groups to look more at bodies than heads. Dwell time to these interest areas were, perhaps not unexpectedly, highly correlated, although neither were correlated with dwell time to the background, suggesting that fixations not being allocated to heads were being redirected to bodies, rather than to non-social elements of the scene in these groups. This reaffirms the notion that social objects are more attention capturing than non-social objects, regardless of group, in this experiment. However, it is difficult to ascertain from the results of the current study, whether participants who were viewing the “live” webcam were purposely allocating additional attention to the bodies of the actors because they were more salient stimuli for these groups or whether they were specifically avoiding looking at faces. There are both social and non-social explanations which might account for the increased attention to bodies in our social conditions. In the former category, if the social rule of not staring at others were responsible for the lack of attention to heads in this group, fixating the body instead might allow for an optimal position from which to attend to heads of the actors, covertly, in parafoveal vision. However, as will be discussed more thoroughly in the next section, the social groups demonstrated reduced gaze orienting compared to the free-viewing group, a finding which is contrary to the idea that participants were covertly attending to heads. In addition, if social rules really were governing gaze allocation in the social groups because of a suspicion that the actors could view the participants, one would also expect that the equally or perhaps even more socially unacceptable behaviour of staring at women’s bodies would also be avoided. A possibly more convincing and non-social interpretation of this finding is suggested by recent research which has demonstrated that a greater proportion of time is spent fixating bodies when passively viewing dynamic scenes containing multiple characters relative to static scenes containing single characters [61]. These authors attributed this effect to the participants looking away from the heads of the actors in order to reduce cognitive load associated with processing multiple moving faces. Whilst this would still reflect gaze avoidance rather than attraction to bodies per se, it is possible that given the additional cognitive load that a social context might exert on the participants in the engaged and non-engaged groups, gaze was diverted away from the faces of the actors to their bodies to reduce cognitive load.

### Social Context and the Gaze Orienting Effect

Not only did the participants in the free-viewing condition attend more to heads than those in the social groups, they also showed a significantly pronounced gaze orienting effect relative to the other groups. This was the case for both overt gaze following as measured by saccades made from heads to gaze targets during the shifts and by the more indirect measure of gaze-induced dwell time to targets during and after the gaze shift. It is interesting to note that only the former of these measures correlated to dwell time to heads. There was no such correlation between dwell time to gaze targets and to heads. This suggests that the gaze of the actors was biasing attention to the targets of their gaze even without the need to first fixate on the head. Therefore participants must have been noticing the shifts covertly. This highlights an interesting phenomenon which would not have been evident had we analysed only saccades from heads to targets.

The gaze orienting effect has variously been described as being “reflexive” [19,62,63] and “automatic” [24,64,65] in standard gaze cueing studies, as it appears to occur with such robustness and reliability. It is important to note that in such studies, the way that gaze following is operationalised is very rarely by examining overt, spontaneous saccade generation or fixations to cued targets as we have reported. Rather it is usually defined as small differences in saccadic reaction times to look at cued and uncued targets, typically found to be in the region of 30ms advantages for cued targets. However, it was striking that in our study, overt gaze following rates were low: only 10% of the shifts were overtly followed in the social groups whilst rates of 37% were found in the free-viewing group. This casts great doubt upon the notion that gaze following is reflexive or obligatory. Particularly when viewed in a rich social context, gaze following seemed anything but obligatory, with as many as 90% of shifts not being overtly followed and less than 10% of fixations falling on the objects being gazed at. In contrast, gaze following behaviours were most evident in the condition which was least like a real social situation and most comparable to the majority of previous studies into social attention; in our free-viewing group. It therefore seems that gaze following only occurs to any degree when a rich social context is absent.

Interpreting the current findings with reference to the extant literature on gaze following in naturalistic scenes provides only one comparable task. In Kuhn et al.’s magic trick task [50,52], participants were found to spend around 20% more time looking at the hand the magician gazed at than at the hand he did not gaze at. This compares to only a 12% dwell time advantage for gazed-at objects in our free-viewing condition, and only 5% in the socially-engaged condition with no significant difference in the non-engaged group. However, participants completing this task did not watch what they believed to be a live social scene. Furthermore, participants were aware that they were watching a magic trick and would be attempting to identify how the trick was achieved and hence cues of the magician would be expected to command higher attentional priority than in our unpredictable social scene. It may not be surprisingly then, that more attention was paid to gazed-at objects in this task (i.e. the magician’s hands) than the targets in our scene, given these differences in scene content and viewing context.

But why might the social context reduce gaze following relative to when free-viewing the scene? The absence of attentional bias to heads in the social groups may explain why participants in these groups overtly followed the gaze shifts less: participants were noticing the shifts to a lesser degree as they were fixating the actors’ heads less often. However, dwell time to gazed-at targets was not related to dwell time to heads, suggesting that even covertly, the social group were not noticing or responding to the gaze shifts as much as those in the free-viewing group. We suggested in the previous section that cognitive load might have been greater in the social groups, which could explain the avoidance of the actors faces in these conditions. The additional cognitive load involved in viewing a live social scene may also explain the more global pattern of reduced responses to gaze cues in the social conditions. In these groups, cognitive resources may have been divided between, on the one hand, visual processing of the scene and on the other hand, more higher level social cognitions related to observing others in “real-time”, which would not have been the case in the free-viewing group.

In further support of the present findings, two observational studies which have examined natural gaze behaviour in social environments have shown that gaze following, far from occurring in a reflexive manner, appears to only operate when the gazer is not facing the observer, that is when the observer responds to the head orientation of the gazer from behind them and when they therefore cannot see their eyes. When walking towards the gazer, observers in fact tend to look away from the gazed at location [66,67]. This finding and that of the current study calls into question the validity of the suggestion that gaze orienting is a “reflexive” behaviour. If gaze following only occurs reliably under standard laboratory conditions and is dramatically reduced or even eliminated by embedding it within a rich social context, then it may be that some previous work has oversimplified and misinterpreted the salience of eye gaze in natural environments and at the same time underestimated the influence of competing influences on cognitive processes in the real-world.

### Limitations

It might be argued that one limitation of the current study is that if one wishes to examine naturalistic social attention, this should be done in a real-time social interaction, not by passively watching actors on a computer screen whilst sitting in a laboratory with head stabilised by a chinrest. Certainly, investigations into “live” social attention will be a welcome addition to present literature, and thanks to mobile eye tracking devices this is becoming increasingly possible. There are however disadvantages to using such systems. Firstly, the experimenter loses a good deal of control over what the participant sees and each participant will view a slightly different stimulus as a consequence. In addition, under such circumstances it would be difficult to enact accurately-timed gaze shifts which were identical for all participants such as those in the current study. Thirdly, data sampling rates are far lower in currently available mobile systems than in desk mounted systems (60Hz is a typical sampling rate for mobile systems, as opposed to the 1000hz possible with static eye trackers) meaning that analysis of eye movement metrics will be far less fine-grained than those possible with static systems, which may result in failure to detect subtle differences in patterns of gaze allocation. We suggest that providing a social context within the fixed-eye tracking lab as we have done in the current study, provides a credible methodology for examining social attention in real-time whilst retaining the spatial and temporal accuracy of stimulus presentation and data analysis which is critical to traditional experimental designs.

A further potential limitation relates to the instructions given to participants in the different conditions in this experiment. It is readily acknowledged that different task instructions were given to the participants in the social groups compared with the free viewing group. The reason for this was that we wanted to create a plausible social context and this required significant embellishment. We do not deny that these instructions will have influenced the eye movement behaviour of our participants as this was our intention: that the social context would modulate attention to the people in the scene. It is not possible from the current study to quantify the contribution of each individual element of our manipulation to creating the social context. However, we did discover during a pilot of the manipulation that without the second experimenter entering into the lab at the beginning of the task to say that the other participants (the actors) had arrived, participants did not believe that the video was live. It was therefore imperative that we embellished the set up to this extent in order to provide the context we were trying to create. As one anonymous reviewer also pointed out, there were also differences between the two social groups in terms of instructions which may have influenced gaze behaviour: namely, in the engaged group, participants were informed that we wanted them to view the actors because we were interested in how it would affect their group task performance compared to participants who did not get to view the other participants. In contrast, participants in the non-engaged group were just asked to watch the other participants. Whilst we acknowledge that this may have influenced gaze behaviour unrelated to the engaged and non-engaged manipulations, and is something to be considered in future experiments, we are confident that it had little influence on behaviour in this task as eye movement analyses revealed almost identical patterns of social attention in terms of viewing behaviour and gaze following between the two social groups.

### Future Directions

We have provided here an example of a methodology for investigating genuinely “social” social attention under controlled laboratory conditions. Our approach shares similarities with a contemporary movement in the study of cognition: the cognitive ethology approach. Recently advocated by Kingstone and colleagues [11,35–37], cognitive ethology suggests that laboratory-based investigations in cognitive psychology should be complemented with observations obtained in the real-world of natural behaviour to provide a richer and more accurate representation of cognitive processes. Cognitive ethology acknowledges that whilst valuable insights have been gained from traditional laboratory-based cognitive paradigms, by constraining experimental stimuli and behavioural responses within such tasks risks the possibility of misrepresenting or failing to identify important aspects of cognitive function. It further posits that human cognition and attention in particular are situationally dependent, and will change as the participants’ understanding of a situation or the situation itself, changes. The findings of the current study support the assumptions of the cognitive ethology approach by demonstrating the importance of context in attention allocation within more naturalistic social scenes. Recent technological advances in mobile eye tracking have allowed some authors to fully adopt the cognitive ethology approach in their social attention research by examining eye movement behaviour within naturalistic settings and real-world social interactions, although these advances do come at a cost, as described in the previous section. Nevertheless, future work based on the current study might consider contrasting findings from data collected using static eye trackers and videoed scenes with those obtained from using mobile eye tracking methodology, where participants are physically present within the social scenario and where the potential for a social exchange is very possible. Using such a task would identify whether the physical presence of a potential social partner further modulates social attention or whether it is the belief in a future interaction which is critical in altering the social attention observed here.

Recent work [54] has shown that gaze behaviour during an experimenter-initiated social exchange produces a different pattern of gaze allocation compared to studies observing eye movements in response to non-interacting strangers [49,55]. It may not be surprising to find that more eye contact and gazes toward an individual’s face occur when people are engaged in a conversation with that person than when they walk past a stranger in the street. This contrast serves to highlight the important role of context and experience in influencing social attention behaviour. Many implicitly learned social rules are likely to govern how we attend to social others in the real world, and it is imperative that future work begins in earnest to disentangle this complex web of factors which impinge on how we perceive those around us if we are to truly understand naturalistic human behaviour.

## Conclusion

Previous work on social attention has failed to account for the influence of situational context on social attention. The current study sought to assess the role of social context when viewing a dynamic social scene in modulating social attention and gaze following. Rather than increasing attention to heads and enhancing the gaze following mechanism, participants who believed they were watching a live webcam of other participants followed gaze less and attended less to the heads of the people in the scene than participant who believed they were watching a pre-recorded video, who showed a more “typical” patterns of social attention when compared to previous studies. There were very few differences between the socially engaged (social interaction imminent) and the non-engaged groups (interaction not imminent) in their attention allocation to the scene suggesting that the belief that the protagonists were real and in close proximity was enough to attenuate both attention to heads and the gaze following mechanism, findings which may reflect increased cognitive load in the social groups. Acknowledging that the social context has an important role to play in social attention studies may better inform future research in this area by encouraging researchers to consider real-life factors in social attention processes.

## Supporting Information

S1 Dataset Mean proportion dwell time to interest areas over the whole trial. (SAV)

S2 Dataset Mean proportion dwell time to gaze targets before and after each gaze shift. (SAV)

S3 Dataset Mean proportion of gaze shifts overtly followed, as defined by shifts where a saccade started in the head IA of the gazing actor and landed in the target IA. (SAV)
